# Intra-strain genomic microevolution and phage resistance in *Pseudomonas aeruginosa* PAO1 laboratory isolates

**DOI:** 10.1128/spectrum.03299-25

**Published:** 2026-07-21

**Authors:** Ayaka Washizaki, Ajeng K. Pramono, Arata Sakiyama, Keiko Inaba-Hasegawa, Shoichi Mitsunaka, Hiroki Ando

**Affiliations:** 1 Laboratory of Phage Biologics, Graduate School of Medicine, Gifu University 12785, Gifu City, Gifu, Japan; 2 Center for One Medicine Innovative Translational Research (COMIT), Institute for Advanced Study, Gifu University 12785, Gifu City, Gifu, Japan; 3 Venture Unit Engineered Phage Therapy, Discovery Accelerator, Astellas Pharma Inc. 714925 , Tsukuba City, Ibaraki, Japan; 4 Arrowsmith Inc., Tsukuba City, Ibaraki, Japan; CEB - Centre of Biological Engineering, Universidade do Minho, Braga, Portugal

**Keywords:** *Pseudomonas aeruginosa*, phage PP7, PAO1, microevolution

## Abstract

*Pseudomonas aeruginosa* is a major opportunistic pathogen, and its laboratory reference strain, PAO1, is widely used in microbiological and genetic studies. However, PAO1 often exhibits phenotypic variability that can affect experimental reproducibility. Our PAO1 stock, obtained from a public biobank, is resistant to PP7, a pilus-dependent single-stranded RNA phage known to infect PAO1. This suggests the presence of genetic variants in the stock. To check this possibility, we isolated six phenotypically distinct variants (GU1–GU6) and performed genomic and phenotypic analyses. Notable differences were observed among the isolates in terms of motility, pyocyanin production, and susceptibility to PP7. Whole-genome sequencing revealed that four of the six variants harbored mutations in pilus-associated genes. Among these, GU3 carried a mutation in *pilT*, which encodes a motor protein essential for type IV pilus retraction, and the loss of retraction led to the PP7 resistance. GU2, GU4, and GU6 shared a nonsense mutation in *pilJ*, a gene involved in chemotaxis and pilus extension, resulting in reduced twitching motility and lower PP7 infection efficiency. Additionally, we found that a mutation in *lasR*, a master regulator of quorum sensing, promoted the replication of prophage Pf6, which was integrated into the PAO1 genome. Pf6 replication interferes with PP7 infection, providing an alternative mechanism of resistance. These findings offer new insights into the complexity of phage-host interactions and emphasize the importance of routine verification and careful handling of *P. aeruginosa* sublines used in bacteriological and phage research.

**IMPORTANCE:**

Phenotypic and genotypic variability in *Pseudomonas aeruginosa* PAO1 has been widely reported, raising concerns regarding the reproducibility of laboratory studies that rely on this reference strain. In this study, we isolated six PAO1 variants from a single laboratory stock and demonstrated that they differed markedly in motility, pyocyanin production, and susceptibility to the ssRNA phage PP7. Whole-genome sequencing has revealed that even a single mutation in a pilus-associated gene can profoundly affect bacterial motility and phage susceptibility. Furthermore, we showed that a mutation in *lasR*, a key regulator of the quorum-sensing system, triggered replication of the Pf6 prophage, which in turn hindered PP7 infection. These findings underscore the dynamic nature of laboratory strains and highlight the need for caution when interpreting results from phage-host interaction studies using reference strains. Our results provide a new understanding of how subtle genetic changes in model strains influence experimental outcomes in microbiology.

## INTRODUCTION

*Pseudomonas aeruginosa* is a Gram-negative bacterium commonly found in diverse environments and is a major opportunistic pathogen, particularly in immunocompromised individuals. In patients with cystic fibrosis, *P. aeruginosa* establishes chronic lung infections by adapting to an environment through mutations that enhance survival against host immune defenses and antibiotics (1–4).

Standardized laboratory strains are essential for understanding these pathogenic mechanisms and developing effective strategies for treating *P. aeruginosa* infections. *P. aeruginosa* PAO1, one of the most widely used reference strains, is a derivative of PAO (originally called “*P. aeruginosa* strain 1”), which was isolated in 1954 from a wound in Melbourne, Australia (5, 6). PAO acquired chloramphenicol resistance through spontaneous mutation, resulting in the derivative strain PAO1. Over time, various PAO1 sublines have been distributed and maintained globally; for example, ATCC 15692 was deposited by Holloway to the American Type Culture Collection (ATCC).

However, similar to the clinical isolates, PAO1 undergoes microevolution during laboratory cultivation and storage. Klockgether et al. (7) reported the genomic and phenotypic divergence among PAO1 strains maintained in different laboratories, affecting traits such as nutrient utilization and virulence in an acute murine airway infection model. More recently, Chandler et al. (8) also reported genomic and phenotypic diversity among 10 PAO1 sublines. These studies demonstrate that PAO1 strains accumulate mutations during laboratory handling, leading to functional differences that may compromise reproducibility across studies, even when strains are sourced from the same distributor.

We obtained the ssRNA phage PP7 and a PAO1 strain from the Biological Resource Center, National Institute of Technology and Evaluation (NBRC). PP7 is an ssRNA phage originally isolated from sewage in Pangbourne, England (9). PP7 uses type IV pili (TFP) as a receptor for adsorption and binds to the β1–β2 loop in the variable region 2 of PilA. After binding to PilA, PP7 reaches the host cell surface by pilus retraction and establishes infection (10–13). Although NBRC lists PAO1 (NBRC 106052, hereafter referred to as PAO1-N) as a host for PP7, it also provides the “Information for Users” as “Cultures lose phage sensitivity after a few months at room temperature. Two colony (smooth or rough) types may appear. Their 16S rRNA sequences are identical.” (https://www.nite.go.jp/nbrc/catalogue/NBRCCatalogueDetailServlet?ID=NBRC&CAT=00106052). This implies that PAO1-N stocks consist of genetically distinct clones, some of which may harbor mutations that affect PP7 sensitivity.

In this study, we aimed to determine whether PAO1-N stocks consist of genetically distinct clones and systematically characterize the genomic and phenotypic diversity of PAO1-N-derived isolates. We also sought to identify genetic alterations responsible for changes in sensitivity to the phage PP7. To this end, we isolated six PAO1-N-derived variants and characterized their phenotypic traits. Comparative genome analysis of the six variants and PAO1 obtained from ATCC (ATCC 15692, hereafter referred to as PAO1-A), together with further phenotypic analysis, revealed that mutations in genes associated with pilus function affect phage sensitivity.

Our findings highlight the extent to which laboratory strains of *P. aeruginosa* diverge over time, both genotypically and phenotypically, even when sourced from the same reference stock. Such microevolutionary changes can obscure reproducibility and complicate studies involving phage-host interactions. Despite the widespread use of PAO1 as a standardized model strain, its genetic instability raises important concerns regarding its experimental consistency, including that in phage studies.

## RESULTS

### PP7 susceptibility of PAO1-N and isolation of its variants

We obtained *P. aeruginosa* PAO1 strains from both the NBRC and ATCC, which were originally isolated in 1954 and were maintained independently. PAO1 is a well-established host for the phage PP7, and this PAO1-PP7 pairing has been widely used in previous studies (13–16). However, NBRC mentioned on the website that “cultures lose phage sensitivity after a few months at room temperature” (https://www.nite.go.jp/nbrc/catalogue/NBRCCatalogueDetailServlet?ID=NBRC&CAT=00106052).

**Fig 1:**
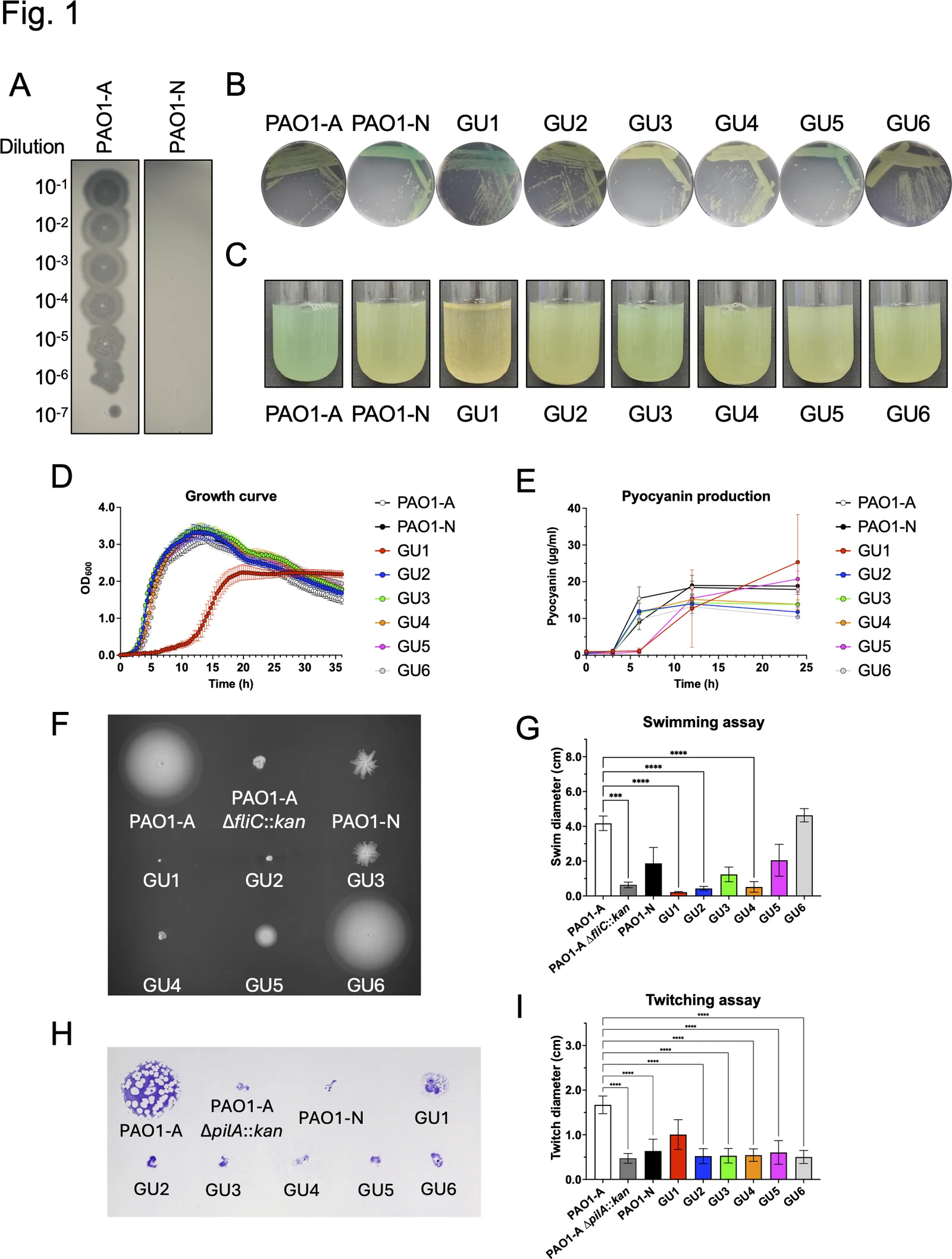
Phenotypic differences of GU variants. (**A**) PP7 was serially diluted and spotted on a lawn of PAO1-A or PAO1-N. Plates were incubated at 37°C for 7.5 h. (**B**) Colony morphology was observed by streaking from the glycerol stock onto a plate and incubated overnight at 37°C. (**C**) Coloration of liquid culture was analyzed by inoculating bacteria from glycerol stocks and incubating them overnight at 37°C with shaking at 250 rpm. (**D**) Bacteria were inoculated into lysogeny broth (LB), and the OD_600_ was measured every 20 min until 36 h. The experiment was performed in triplicate and repeated three times. The mean of three experiments and SD were plotted. (**E**) Pyocyanin was extracted at 3, 6, 12, and 24 h and measured using *A*_520_. Experiments were performed in triplicate and repeated three times. Mean and SD are provided. (**F and G**) To analyze swimming motility, a single colony was picked and touched onto a 0.25% agar plate. The swimming diameter was measured after overnight incubation at 30°C. Experiments were performed in triplicate and repeated three times. (**F**) Representative picture and (**G**) mean and SD are shown. Kruskal-Wallis test was performed for statistical analysis: ***, *P* < 0.001; ****, *P* < 0.0001. (**H and I**) To analyze twitching motility, a single colony was picked and inoculated into an LB 1.0% agar plate. After overnight incubation at 37°C, the agar was removed, and the plates were stained with crystal violet. Experiments were performed in triplicate and repeated three times. (**H**) Representative picture and (**I**) mean and SD are shown. Kruskal-Wallis test was performed for statistical analysis: ****, *P* < 0.0001.

To determine whether the PAO1-N maintained in our laboratory still retained susceptibility to PP7, we performed spot tests. PP7 failed to infect PAO1-N and did not form plaques (Fig. 1A). To exclude the possibility that PP7 obtained from NBRC shows a discrepancy in its phenotype compared to previous reports, the morphology of PP7 was confirmed by transmission electron microscopy (TEM). PP7 lysates maintained in our laboratory showed icosahedral capsids without tails, consistent with previous reports (17) (Fig. S1A). Moreover, to test whether our PP7 infection depends on pili as described in previous reports, we examined PP7 infectivity using PAO1-A mutants defective in pilus, flagellum, or lipopolysaccharide (LPS) synthesis (Fig. S1B). As an additional reference strain, we also tested PA14, which has been reported to be resistant to PP7 (15), and confirmed its resistance (Fig. S1B). PP7 infection of PAO1-A is independent of flagella or LPS but dependent on pili. These results indicate that PP7 maintained in our laboratory retained the same characteristics as those described in previous reports.

The observation of PP7 morphology and infectivity, along with a note from the NBRC stating that “two colony (smooth or rough) types may appear” (https://www.nite.go.jp/nbrc/catalogue/NBRCCatalogueDetailServlet?ID=NBRC&CAT=00106052), supported our concern that the PAO1-N stock might consist of multiple genetically distinct clones. To test this possibility, we randomly picked six single colonies (GU1–GU6) that were subsequently used for phenotypic analysis.

### Phenotypic differences among GU variants

The six PAO1-N-derived GU variants exhibited distinct colony morphologies when streaked onto lysogeny broth (LB) Lennox agar plates. GU1 and GU5 formed rough green colonies, whereas GU2, GU3, GU4, and GU6 produced smooth yellow colonies resembling those of PAO1-A (Fig. 1B). Interestingly, after overnight incubation in liquid culture, GU3 and PAO1-A exhibited green coloration, whereas the other variants appeared yellow. Notably, GU1 showed strong autolysis, resulting in visible cell debris and significantly lower optical density than the other variants (Fig. 1C). Growth curves revealed that all variants except GU1 had growth rates comparable to those of PAO1-A. GU1 grew more slowly and reached a lower OD_600_ plateau, likely because of autolysis. Interestingly, while the OD_600_ of the other variants decreased after 12 h of incubation, GU1 maintained its OD_600_ up to 36 h (Fig. 1D).

The kinetics of pyocyanin production varied among variants. PAO1-A initiated pyocyanin production at 6 h, similar to GU2, GU3, GU4, and GU6. In contrast, GU1 and GU5 exhibited delayed pyocyanin synthesis beginning at 12 h (Fig. 1E).

Motility assays revealed phenotypic differences. In the swimming assay, only GU6 displayed motility comparable to PAO1-A (average swim diameter: 3.75 ± 1.6 cm vs 4.09 ± 1.9 cm), while GU1–GU5 showed reduced motility (Fig. 1F and G). These results suggest that GU1–GU5 have compromised flagellar activity.

In the twitching assay, GU1 showed slightly reduced motility; however, this difference was not statistically significant. Other variants exhibited severely impaired twitching, with significantly smaller diameters than PAO1-A (*P* < 0.0001) (Fig. 1H and I). These results indicate that the phenotypic diversity among the PAO1-N variants arises from distinct genetic alterations that affect motility, pigment production, and growth behavior.

### Drug susceptibility profiles of PAO1-N variants

Previous studies have reported variability in the drug resistance profiles of PAO1 strains maintained in different laboratories (8). To determine whether GU variants acquired unique resistance profiles, we performed MIC testing. All six variants showed drug susceptibilities identical to those of PAO1-A (Table 1). This suggests that the variants were maintained under conditions without drug selection pressure, thereby minimizing the likelihood of resistance-related mutations.

**TABLE 1:** Drug susceptibility of GU variants^*a*^

	MIC (µg/mL)													
Variant	PIP	TZP/PIP	FEP	CAZ	DOR	IPM	MEM	GEN	AMK	TOB	LVX	CIP	CST	ATM
PAO1-A	4	4/4	1	1	1	1	0.5	1	4	1	1	0.25	1	4
PAO1-N	4	4/4	1	1	1	1	0.5	1	4	1	1	0.25	1	4
GU1	2	4/2	1	1	1	1	0.5	1	4	1	0.5	0.25	1	2
GU2	4	4/4	2	2	1	1	0.5	1	4	1	0.5	0.25	1	4
GU3	4	4/4	1	2	1	1	0.5	1	4	1	0.5	0.25	1	8
GU4	4	4/4	1	2	1	1	0.5	1	4	1	0.5	0.25	1	4
GU5	4	4/4	1	2	1	0.5	0.5	1	4	1	1	0.25	1	4
GU6	4	4/4	1	2	1	1	0.5	1	4	1	1	0.25	1	4
GU7	4	4/4	1	2	1	1	0.5	1	4	1	0.5	0.25	1	4

^*a*^ The MIC was measured using premade dry plates (DP-45), according to the manufacturer’s instructions. PIP, piperacillin; TZP, tazobactam; FEP, cefepime; CAZ, ceftazidime; DOR, doripenem; IPM, imipenem; MEM, meropenem; GEN, gentamicin; AMK, amikacin; TOB, tobramycin; LVX, levofloxacin; CIP, ciprofloxacin; CST, colistin; and ATM, aztreonam.

### PP7 susceptibility profiles of PAO1-N variants

**Fig 2:**
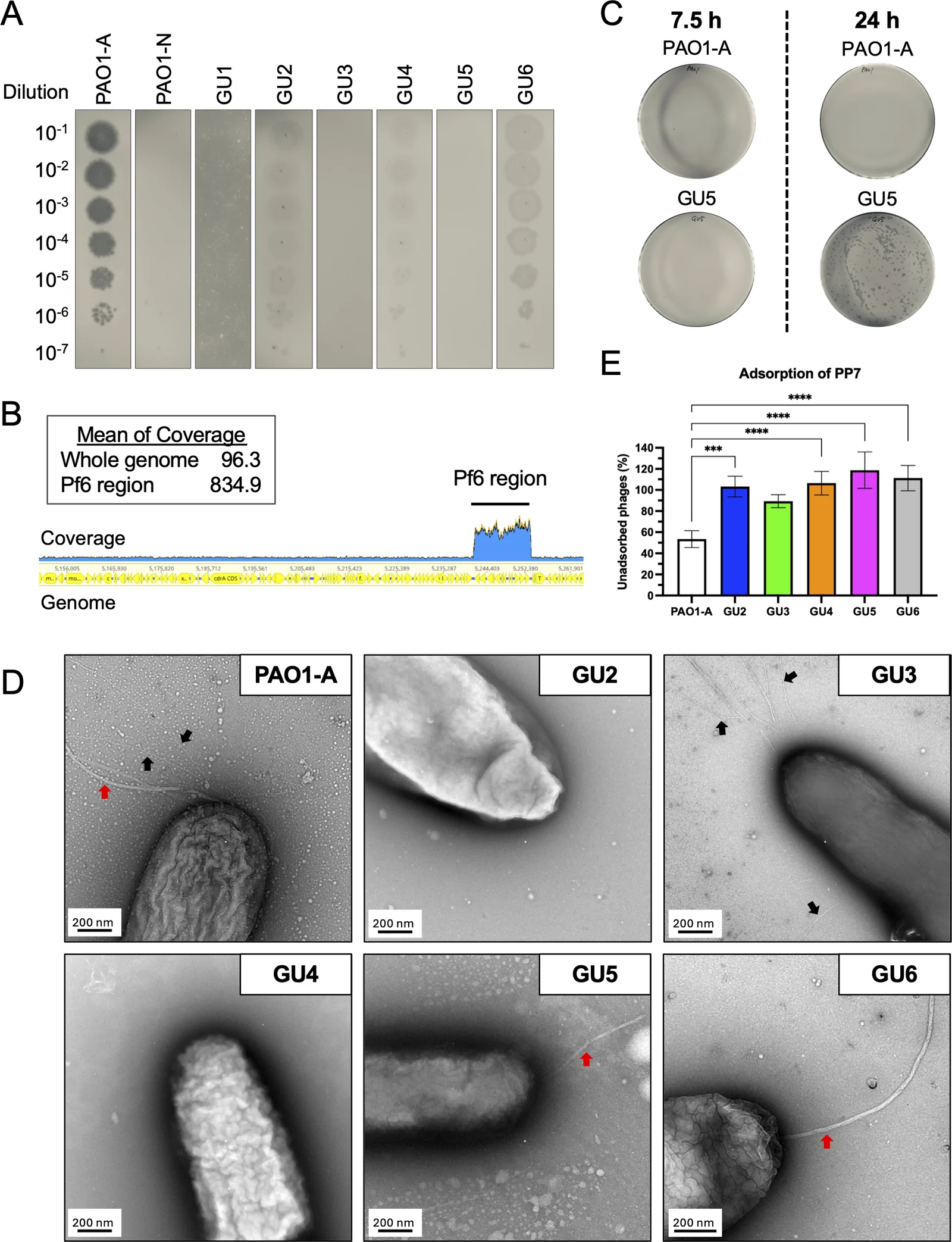
PP7 sensitivity, adsorption, pf6 replication, and cell morphology observed by TEM. (**A**) PP7 was serially diluted and spotted on the lawn of variants shown in the figure. Plates were incubated at 37°C for 7.5 h. (**B**) Coverage of Pf6 prophage region of GU5 is shown. (**C**) To observe autoplaquing of GU5, the bacteria indicated in the graph were mixed with LB 0.4% soft agar and plated on LB plates. Plates were incubated for 7.5 or 24 h at 37°C. (**D**) Bacterial culture was negatively stained with uranyl acetate and visualized by JEM-2100. Black arrows indicate the pilus, and red arrows indicate the flagellum. Scale bar, 200 nm. (**E**) PP7 adsorption assays were performed using PAO1-A and the GU variants. Host bacteria were cultured to approximately 5 × 10^8^ CFU/mL and concentrated 20-fold. PP7 was added at a multiplicity of infection of 0.001. At 10 min post-infection, the titer of unadsorbed phages was measured. The percentage of unadsorbed phages was calculated by setting the phage titer in the sample without host bacteria to 100%, and mean and SD are shown. Experiments were performed in triplicate and repeated three times. Kruskal-Wallis test was performed for statistical analysis: ***, *P* < 0.001; ****, *P* < 0.0001.

Although the NBRC website describes PAO1-N as a host for PP7, it showed no susceptibility to PP7 in our laboratory (Fig. 1A). This raises the possibility that PP7-resistant variants within the PAO1-N stock interfere with plaque formation. To investigate this hypothesis, we performed plaque assays on individual GU variants. GU1 could not be evaluated because of its strong autolysis, which prevented lawn formation (Fig. 2A). Therefore, GU1 was omitted from further analyses. GU3 and GU5 were completely resistant to PP7, and no plaques were observed. In contrast, GU2, GU4, and GU6 exhibited partial susceptibility, as indicated by the faint and turbid plaques (Fig. 2A). These results suggest that while PP7-susceptible variants are present in the PAO1-N stock, the presence of resistant clones, such as GU3 and GU5, may hinder plaque formation, giving a false impression of complete resistance in the mixed population.

### Comparative genomics of PAO1-N variants reveals phage resistance mechanisms

To identify genetic factors contributing to PP7 resistance, we performed whole-genome sequencing of the PAO1-A and GU2–GU6 variants. Single nucleotide polymorphisms (SNPs) were called with a frequency cutoff of 80%, and mutations in the coding sequences (CDSs) were analyzed (Table 2).

**TABLE 2:** SNPs found in GU variants^*a*^

	locus_tag	Encoded product	Position in CDS	Polymorphism type	Nucleotide change	Amino acid change	Protein effect
GU2	BGV84_RS14605	LysR family transcriptional regulator	376	SNP	C → T	D → N	Substitution
	BGV84_RS18035	Flagellar biosynthesis protein FlhA	247	Deletion	GGG → GG		Frameshift
	BGV84_RS02100	Chemotaxis chemoreceptor PilJ	1741	SNP	C → T	Q → stop	Truncation
	BGV84_RS22125	Exodeoxyribonuclease V subunit gamma	2002	SNP	G → C	L → V	Substitution
	BGV84_RS24255	Mks condensin complex protein MksB	950	Substitution	CG → AC	P → H	Substitution
GU3	BGV84_RS15050	Hypothetical protein	14	Insertion	CCCCCC → CCCCCCC		Frameshift
	BGV84_RS19830	Transcriptional regulator FleQ	1074	SNP	C → A	W → C	Substitution
	BGV84_RS02020	Type IV pilus twitching motility protein PilT	562	Deletion	−GCGCTGCGCTCG	ALRS →	Deletion
	BGV84_RS24255	Mks condensin complex protein MksB	949	Insertion	CCG → AACAC		Frameshift
	BGV84_RS30005	Type VI secretion system-associated FHA domain protein TagH	63	Insertion	+G		Frameshift
GU4	BGV84_RS14605	LysR family transcriptional regulator	376	SNP	C → T	D → N	Substitution
	BGV84_RS18035	Flagellar biosynthesis protein FlhA	247	Deletion	GGG → GG		Frameshift
	BGV84_RS02100	Chemotaxis chemoreceptor PilJ	1741	SNP	C → T	Q → stop	Truncation
	BGV84_RS24255	Mks condensin complex protein MksB	950	SNP	C → A	P → Q	Substitution
GU5	BGV84_RS13910	3-methyl-2-oxobutanoate dehydrogenase (2-methylpropanoyl-transferring) subunit alpha	875	SNP	C → T	R → H	Substitution
	BGV84_RS18145	Transcriptional regulator LasR	676	SNP	C → T	V → I	Substitution
	BGV84_RS26000	Phosphodiesterase DipA	209	Deletion	−T		Frameshift
	BGV84_RS26000	Phosphodiesterase DipA	201	Deletion	−CA		Frameshift
	BGV84_RS26000	Phosphodiesterase DipA	206	Deletion	−TG		Frameshift
	BGV84_RS29660	PP2C family serine/threonine-protein phosphatase	883	Deletion	CCC → CC		Frameshift
GU6	BGV84_RS00005	Chromosomal replication initiator protein DnaA	4	Insertion	+TCCGTG	→ SV	Insertion
	BGV84_RS02100	Chemotaxis chemoreceptor PilJ	1741	SNP	C → T	Q → stop	Truncation
	BGV84_RS14605	LysR family transcriptional regulator	376	SNP	C → T	D → N	Substitution

^*a*^ Variant SNPs were called with a frequency cutoff of 80%, and the SNPs in the CDS were selected and listed.

Given that PP7 utilizes TFP for adsorption (9, 10, 12), we focused on mutations in pilus-related genes. GU3 harbors a 12-bp deletion in *pilT,* which encodes a motor protein essential for pilus retraction. Loss of PilT function results in hyperpilation and impaired twitching motility (18–22). GU2, GU4, and GU6 share a nonsense mutation in *pilJ*, which is a chemoreceptor gene involved in chemotaxis (20, 23–25). This mutation likely disrupts pilus assembly (26), which is consistent with the observed loss of twitching motility and reduced PP7 susceptibility. Interestingly, GU5 did not have mutations in the pilus-related genes. However, the sequencing coverage of the Pf6 prophage region was significantly higher than that of other regions (Fig. 2B; Table S1), suggesting active replication of prophage Pf6 in GU5. This aligns with the observation of autoplaque formation after 24 h of incubation (Fig. 2C). Recent studies on Pf4, another temperate phage of PAO1, have shown that the capsid protein, PfsE, binds to PilC and inhibits pilus assembly. This prevents superinfection of phages that use the pilus as a receptor (27, 28). Pf6 has a PfsE homolog with a single amino acid substitution. Therefore, the PsfE homolog might have a similar function in preventing PP7 infection.

These results suggest that GU variants harbor mutations in genes that directly or indirectly affect pilus synthesis or function, thereby reducing PP7 adsorption and consequently dampening its infectivity.

### TEM-based analysis of surface appendages in PAO1-N variants

The results obtained from next-generation sequencing (NGS) suggested that the GU variants either failed to elongate the pili or expressed defective pili. To investigate whether the surface morphology of the GU variants was consistent with that expected from the NGS results, pili and flagella were observed by TEM. The absence of both pili and flagella in GU2 and GU4 was consistent with the mutations in *pilJ* and *flhA*. GU6, which also carried a *pilJ* mutation but retained an intact *flhA*, exhibited flagella but not pili (Fig. 2D). This was consistent with the results of the motility assay, which showed that GU2 and GU4 were unable to swim or twitch, whereas GU6 exhibited swimming ability. GU3 displayed pili, but lacked flagella, correlating with a mutation in *fleQ*, a key regulator of flagellar synthesis (Fig. 2D). GU5 possessed flagella but non-visible pili, despite the absence of mutations in pilus-related genes (Fig. 2D). As described above, this phenotype may be explained by the active replication of Pf6, which inhibits pilus assembly via superinfection exclusion mechanisms. These morphological observations were consistent with the results of the motility assay and NGS data, supporting the hypothesis that specific genetic alterations directly affect surface appendage formation, resulting in PP7 sensitivity in each PAO1-N variant.

### Adsorption efficiency of PP7 on GU variants

To confirm whether phage resistance of the GU variants was associated with reduced PP7 adsorption caused by pilus dysfunction, we performed a phage adsorption assay. Approximately 50% of PP7 particles adsorbed to PAO1-A within 10 min of phage addition. In contrast, adsorption efficiency was significantly reduced in all GU variants except GU3; although the reduction in GU3 did not reach statistical significance, its adsorption efficiency was still lower than that of PAO1-A (Fig. 2E). GU3 exhibited hyperpiliation as described above, suggesting that the apparent decrease in unadsorbed PP7 particles may reflect trapping of phage particles on abundant pili. However, despite this apparent adsorption, infection may not be established because attached phage particles may be unable to reach the cell surface due to impaired pilus retraction. These findings indicate that reduced phage adsorption contributes to PP7 resistance in GU variants, although GU3 may be impaired at a post-attachment step rather than at the initial binding step.

### Functional impact of *pilT* and *pilJ* mutations on PP7 susceptibility

To investigate whether mutations in *pilT* and *pilJ* are responsible for reduced PP7 adsorption and decreased PP7 susceptibility, we performed genetic complementation analyses.

Because co-expression of mutant and wild-type (WT) alleles can cause dominant-negative effects in multimeric proteins, direct plasmid complementation in the original GU variants could complicate the interpretation of the results. This concern is particularly relevant for PilJ, whose variants were recently shown to interfere with WT PilJ function when co-expressed (25). A similar effect may also occur for PilT, which functions as a multimeric ATPase. Therefore, we generated clean deletion mutants, GU3Δ*pilT*::*kan*, GU2Δ*pilJ*, GU4Δ*pilJ*, and GU6Δ*pilJ*, and introduced plasmids expressing the corresponding WT genes.

**Fig 3:**
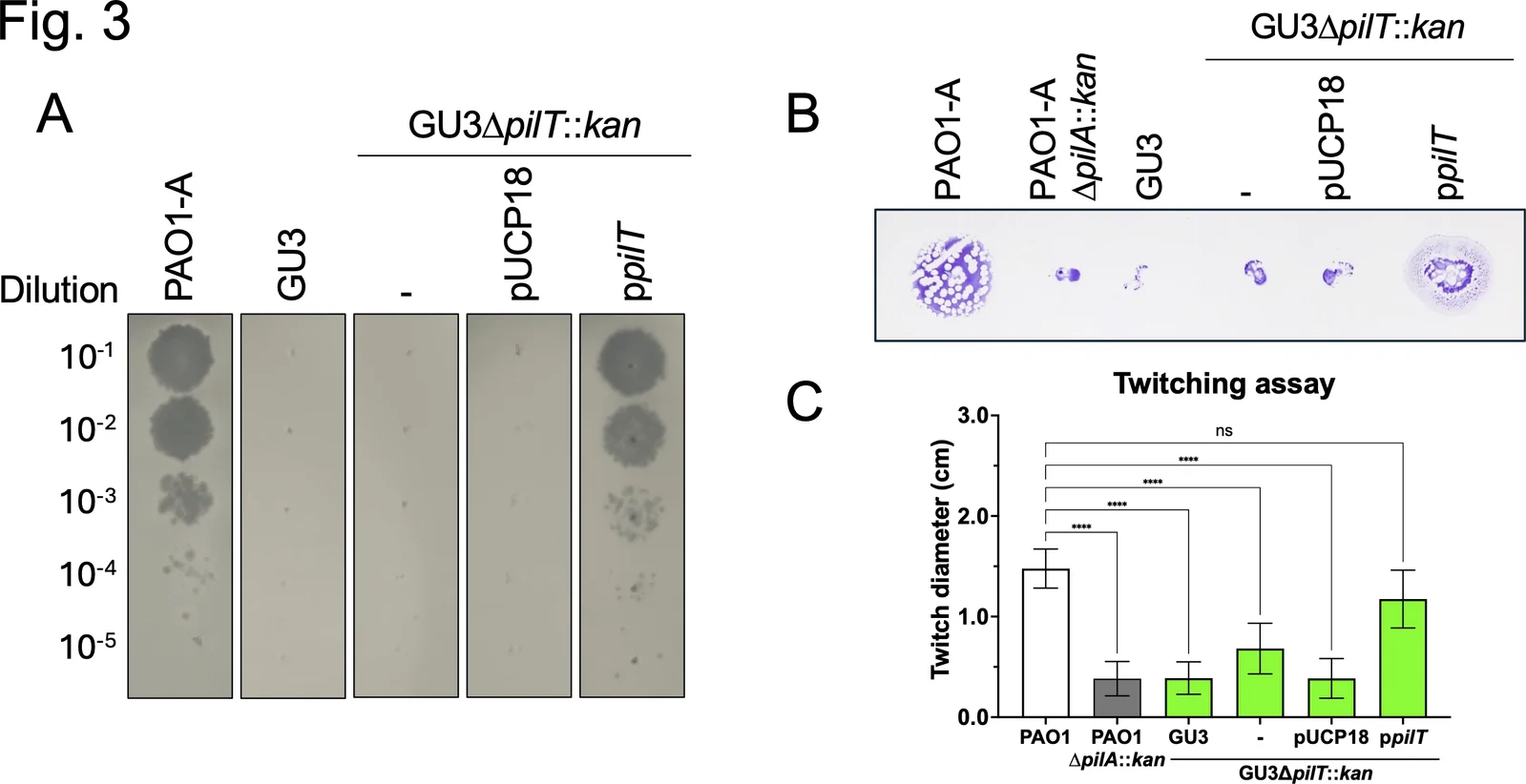
Influences of *pilT* mutation on PP7 infectivity. (**A**) PP7 was serially diluted and spotted on the lawn of variants shown in the figure. Plates were incubated at 37°C for 7.5 h. (**B and C**) To analyze twitching motility, a single colony was picked and inoculated into an LB 1.0% agar plate. After overnight incubation at 37°C, the agar was removed, and the plates were stained with crystal violet. Experiments were performed in triplicate and repeated three times. (**B**) Representative picture and (**C**) mean and SD are shown. Kruskal-Wallis test was performed for statistical analysis: ns, *P* > 0.05; ****, *P* < 0.0001.

Complementation of GU3Δ*pilT*::*kan* with WT *pilT* restored PP7 plaque formation and twitching motility (Fig. 3), demonstrating that the *pilT* mutation is responsible for the PP7 resistance and phenotype of GU3.

**Fig 4:**
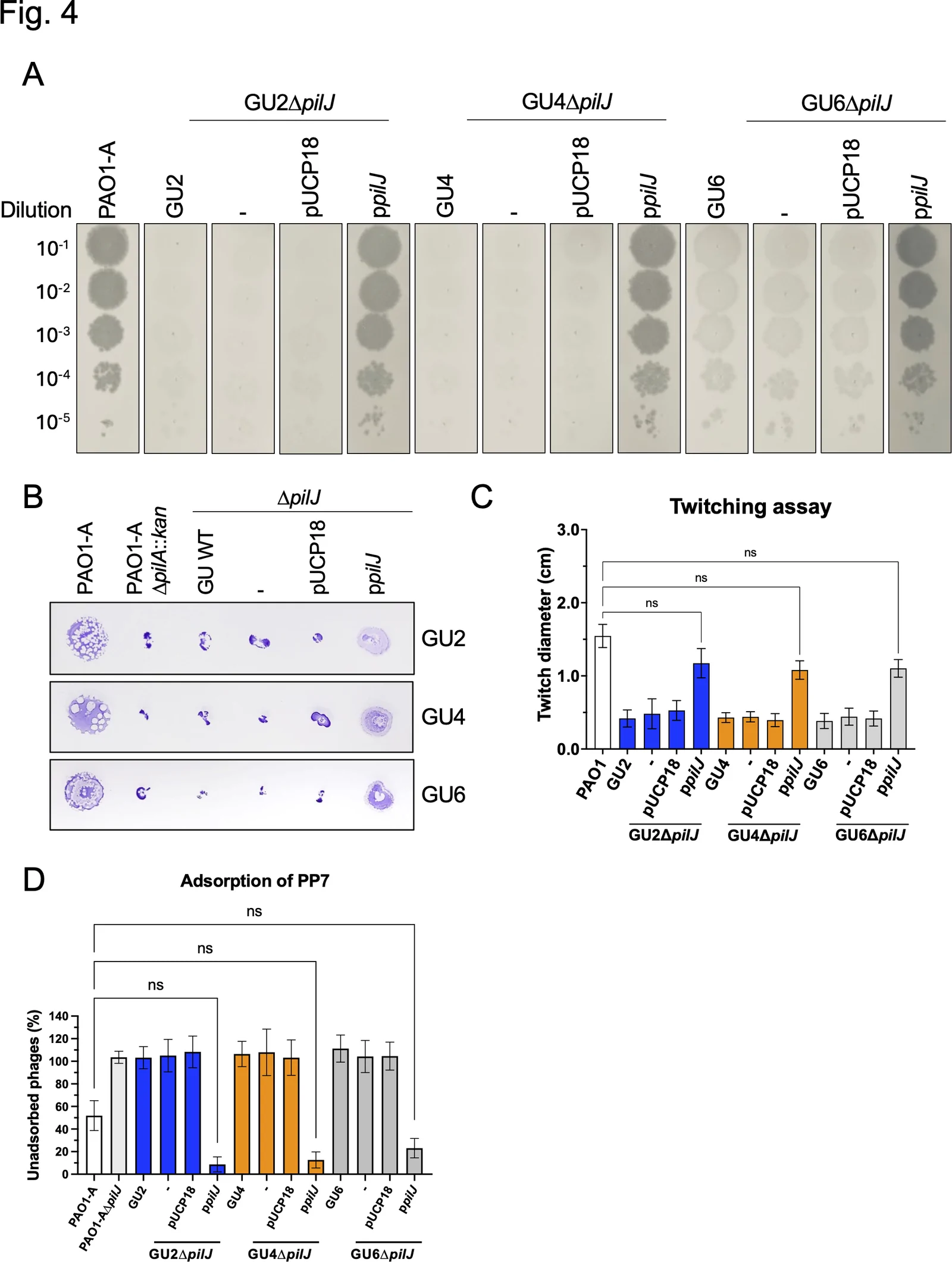
Influences of *pilJ* mutation on PP7 infectivity. (**A**) PP7 was serially diluted and spotted on the lawn of variants shown in the figure. Plates were incubated at 37°C for 7.5 h. (**B and C**) To analyze twitching motility, a single colony was picked and inoculated into an LB 1.0% agar plate. After overnight incubation at 37°C, the agar was removed, and the plates were stained with crystal violet. Experiments were performed in triplicate and repeated three times. (**B**) Representative picture and (**C**) mean and SD are shown. Kruskal-Wallis test was performed for statistical analysis: ns, *P* > 0.05. Without description, the *P* value was less than 0.05. (**D**) PP7 adsorption assays were performed using PAO1-A and the GU variants. Host bacteria were cultured to approximately 5 × 10^8^ CFU/mL and concentrated 20-fold. PP7 was added at a multiplicity of infection of 0.001. At 10 min post-infection, the titer of unadsorbed phages was measured. The percentage of unadsorbed phages was calculated by setting the phage titer in the sample without host bacteria to 100%, and mean and SD are shown. Experiments were performed in triplicate and repeated three times. Kruskal-Wallis test was performed for statistical analysis: ns, *P* > 0.05. Without description, the *P* value was less than 0.05.

Next, the GU2, GU4, and GU6 *pilJ* deletion mutants were complemented with WT *pilJ*. Expression of *pilJ* recovered twitching motility and enabled formation of clear PP7 plaques in GU2Δ*pilJ*, GU4Δ*pilJ*, and GU6Δ*pilJ* (Fig. 4A through C). PP7 adsorption assay showed little or no adsorption to the *pilJ* deletion mutants of PAO1-A, GU2, GU4, and GU6, whereas adsorption was restored by plasmid-borne expression of WT *pilJ* (Fig. 4D). Together, these findings indicate that *pilJ* is required for efficient pilus function and PP7 adsorption.

Collectively, these results demonstrate that proper pilus function is essential for efficient PP7 infection and that mutations in *pilT* and *pilJ* directly alter phage resistance in PAO1-N variants.

### Role of Pf6 and LasR in modulating PP7 susceptibility

**Fig 5:**
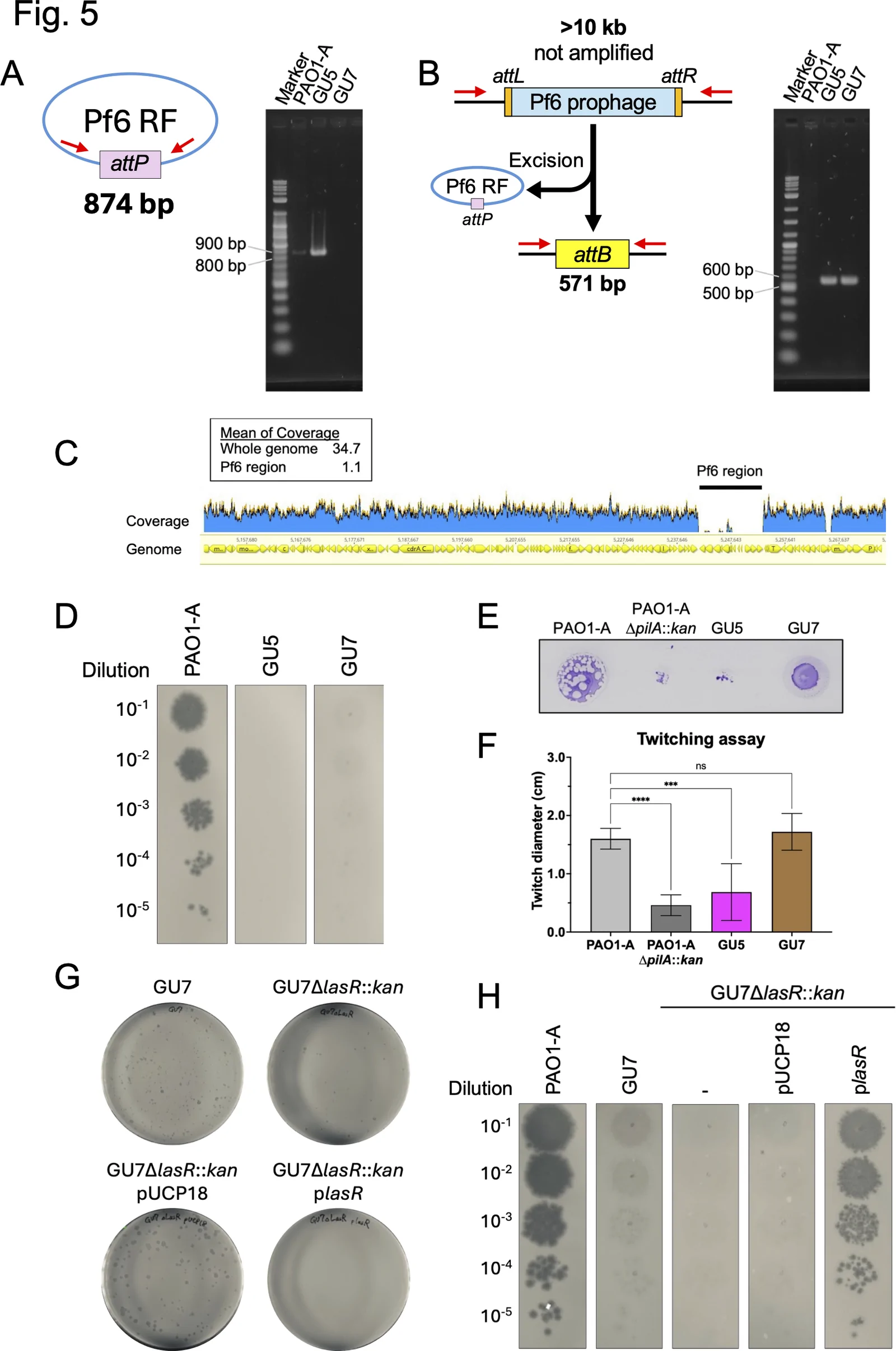
Pf6 replication and PP7 infectivity. (**A**) Pf6 replicative form was detected by amplifying the *attP* site. (**B**) The integration site of Pf6 was amplified by PCR. If Pf6 was integrated, the PCR product would be more than 10 kbp and would not be amplified by the PCR conditions used here. (**C**) Coverage of Pf6 prophage region of GU7 is shown. (**D and H**) PP7 was serially diluted and spotted on a lawn of variants shown in the figure. Plates were incubated at 37°C for 7.5 h. (**E and F**) To analyze twitching motility, a single colony was picked and inoculated into an LB 1.0% agar plate. After overnight incubation at 37°C, the agar was removed, and the plates were stained with crystal violet. Experiments were performed in triplicate and repeated three times. (**E**) Representative picture, and (**F**) mean and SD are plotted in the graph. Kruskal-Wallis test was performed for statistical analysis: ns, *P* > 0.05; ***, *P* < 0.001; and ****, *P* < 0.0001. (**G**) To investigate autoplaquing of GU7, bacteria indicated in the graph were mixed with LB 0.4% soft agar and plated on an LB plate. Plates were incubated for 24 h at 37°C.

Whole-genome sequencing revealed that GU5 showed elevated coverage of the Pf6 prophage region (Fig. 2B; Table S1), suggesting active Pf6 production in GU5. A recent study showed that the PfsE protein of Pf4 inhibits pilus assembly, thereby resulting in superinfection exclusion of pilus-dependent phages (27, 28). Because Pf6 possesses a PfsE homolog, we hypothesized that Pf6 replication inhibits PP7 infection. To check this possibility, we first confirmed Pf6 replication in GU5 by detecting the Pf6 replicative form (RF) using PCR that specifically amplifies Pf6 RF but not Pf4 RF (Fig. 5A; Fig. S2A and B). The amount of Pf6 RF in GU5 was higher than in PAO1-A. We next examined whether a subpopulation of GU5 cells had lost the Pf6 prophage from the genome. To this end, the Pf6 integration site was amplified by PCR. In GU5, a 571-bp band corresponding to the empty Pf6 integration site was observed, whereas this band was not detected in PAO1-A (Fig. 5B; Fig. S2C and D). These results indicate that Pf6 actively replicates in GU5, and that variants lacking Pf6 from the genome are spontaneously generated.

To investigate whether Pf6 replication prevents PP7 infection, we isolated a Pf6-free derivative of GU5. Following streaking on an LB plate, a colony that had spontaneously lost the Pf6 prophage was identified and designated GU7. PCR targeting the Pf6 RF and the Pf6 integration site showed that the Pf6 prophage was absent from the original integration site in GU7 (Fig. 5A and B). To exclude the possibility that the excised Pf6 prophage had reintegrated elsewhere in the GU7 genome, we sequenced the whole genome and confirmed that almost no reads mapped to the Pf6 region (Fig. 5C). Furthermore, PCR using a primer pair targeting an internal region of the Pf6 prophage showed that no amplicon was detected in GU7 (Fig. S2E). Comparison of the genome sequences of GU5 and GU7 revealed that no additional mutations were identified in GU7. These results clearly showed that GU7 is a derivative of GU5 that lost Pf6. GU7 regained its twitching motility and showed partial susceptibility to PP7, forming turbid plaques (Fig. 5D through F). However, persistent autoplaquing and turbid plaques suggested that additional factors were involved in PP7 susceptibility (Fig. 5G). Both GU5 and GU7 carry a mutation in *lasR*, a master regulator of quorum sensing (QS) known to influence prophage replication (29, 30). To investigate the role of *lasR* in PP7 susceptibility, mutated *lasR* was deleted, and WT *lasR* was expressed from a plasmid. Complementation eliminated autoplaquing (Fig. 5G) and restored clear plaque formation by PP7 (Fig. 5H). Furthermore, to determine whether Pf4 was responsible for the autoplaques observed in GU7, we picked single GU7 autoplaques and examined the Pf4 RF by PCR. The Pf4 RF was not amplified by PCR, suggesting that the GU7 autoplaques were not attributable to Pf4 but may have been derived from unknown prophages or autolysis (Fig. S3).

These findings strongly indicate that Pf6-mediated superinfection exclusion and LasR dysfunction synergistically contribute to PP7 resistance in GU5.

## DISCUSSION

PAO1 is a laboratory-adapted reference strain used worldwide. Although PAO1 stocks maintained in different laboratories are derived from the same origin, genetic and phenotypic heterogeneity has been reported (7, 8). In our laboratory, PAO1 obtained from NBRC was unexpectedly resistant to PP7. This observation prompted us to investigate whether PP7 resistance was associated with heterogeneity among PAO1 stock and identify the mutations responsible for the altered PP7 infectivity.

The antibiotic resistance profile did not differ between PAO1-A and the other variants. In contrast, mutations were identified in genes related to pilus synthesis and QS, both of which affect PP7 infectivity. These findings suggest that PAO1-N may have been exposed to selective pressure favoring pilus inactivation. Although silent infection by pilus-dependent phages is a possible explanation, our results did not allow us to determine whether phage infection or another unidentified factor caused the selective pressure toward pilus inactivation in PAO1-N.

In our study, a *lasR* mutation was identified in GU5. *lasR* mutations are often observed in *P. aeruginosa* isolates obtained from patients with cystic fibrosis (CF) (3, 31, 32), wound isolates, and non-infected environments (33). Longitudinal analysis of the respiratory secretions from patients with CF showed that mutations in *lasR* and/or *mucA* first emerged, followed by the accumulation of mutations over time (34), suggesting that *lasR* mutations might induce additional mutations. Moreover, long-term incubation or serial passage of PAO1 results in the emergence of *lasR* mutations (35, 36). In addition to being associated with an increased mutation rate, dysfunction of LasR affects various phenotypes, such as colony morphology and biofilm formation (37, 38). Therefore, even a single mutation in *lasR* can cause inconsistencies in experimental results and compromise reproducibility. Although the factors promoting *lasR* mutations remain unclear, monitoring *lasR* mutations is important to ensure experimental reproducibility.

PilJ is a chemoreceptor of the Pil-Chp system, which is involved in chemotaxis (39–41), and *pilJ* mutations impair pilus extension (26, 39). However, another study using *P. aeruginosa* K, a well-studied laboratory strain, showed that *pilA* expression in a *pilJ* mutant was comparable to that in WT and that the *pilJ* mutant had a detectable amount of PilA at the cell surface, although the amount was lower than that in the WT (24). Consistent with these results, GU2, GU4, and GU6, which have nonsense mutations in *pilJ*, did not have observable pili; however, PP7 formed turbid plaques on these variants. These results indicate that full-length pili are not always essential for PP7 infection. Instead, PP7 may bind to a small amount of PilA at the cell surface and inject its RNA genome. Therefore, PP7 could form plaques on GU2, GU4, and GU6; however, the turbid plaque morphology may be explained by reduced adsorption efficiency due to the loss of full-length pili.

*P. aeruginosa* isolates are often infected with temperate filamentous phages (Pf phages) (42, 43). Our PAO1-A strain harbored at least Pf4 and Pf6, but replication of these phages appeared to be tightly controlled. Notably, Pf4 RF was not detected by PCR, and Pf6 RF was detected only at a low level (Fig. S2). In contrast, Pf6 replication was elevated in GU5, and Pf4 replication was not detected in GU5 (Fig. S2). Active Pf6 replication may result in the expression of a PfsE homolog, which could impair pilus synthesis. Previous research showed that PfsE of Pf4 prevents infection by pilus-dependent phages (27). These results suggest that the replication of Pf4 and Pf6 is finely balanced and that disruption of this balance may affect the infectivity of pilus-dependent phages.

The QS system of *P. aeruginosa* consists of three systems, Las, Rhl, and PQS, which regulate the expression of hundreds of genes (44). Previous reports showed that QS mutations induce autolysis or autoplaque formation, suggesting that prophage replication is regulated by the QS system (45, 46). Indeed, PfsE not only affects pilus synthesis but also inhibits PQS signaling and promotes Pf4 replication (29). In addition, Schmidt et al. (47) reported that the deletion of Pf6 from PAO1 upregulated the Las system. Thus, a cellular state with reduced PQS and Las signaling may be permissive for Pf phage replication. In GU5, the *lasR* mutation may have established such a state, leading to enhanced Pf6 replication and disruption of the finely balanced replication of Pf4 and Pf6. This cascade may have ultimately contributed to PP7 resistance, possibly through impaired pilus synthesis mediated by the Pf6 PfsE homolog. In this study, we could not analyze the function of the Pf6 PfsE homolog or the relationship between Pf6 and Las signaling. Further investigations are required to elucidate the complex interplay between Pf phages and the QS system in *P. aeruginosa*.

*P. aeruginosa* obtains various mutations and adapts to harsh environmental conditions. This is also true under laboratory conditions, and the microevolution of *P. aeruginosa* is ongoing. In this study, we isolated and characterized at least six variants of PAO1-N stock that exhibited distinct phenotypes. Of note, this study was not designed to provide a comprehensive or longitudinal analysis of PAO1 microevolution. Instead, our findings represent a case-based characterization of heterogeneity within a PAO1-N stock and its functional consequences for PP7 susceptibility. Therefore, we have limited the discussion of broader PAO1 evolutionary dynamics to a minimum. Nevertheless, our results raise concerns regarding experimental reproducibility and the potential indirect effects of spontaneous mutations on experimental outcomes. These findings indicate that not only PAO1 but also other bacterial strains should be maintained and handled with great caution, and special attention should be paid to potential genetic and phenotypic changes under storage or experimental conditions.

## MATERIALS AND METHODS

### Bacterial strains and growth conditions

*P. aeruginosa* PAO1 strains (GenBank accession number NC_002516.2) were acquired from the Biological Resource Center, National Institute of Technology and Evaluation (NBRC; catalog #NBRC 106052) and American Type Culture Collection (catalog #15692). All strains were preserved in 20% glycerol and 50% lysogeny broth Lennox (Formedium, Swaffham, UK) at −80°C until further use. All strains and isolates were cultivated in LB at 37°C in a MaxQ 4000 orbital shaker (Thermo Scientific, Waltham, MA, USA) or on LB 1.5% agar plates, unless otherwise stated.

### Isolation of six variants from PAO1-N

PAO1-N was streaked onto an LB plate and incubated overnight at 37°C. Six colonies were randomly picked from PAO1-N and named GU1–GU6. These six GU variants were deposited in NBRC under the following NBRC numbers: NBRC 116673, PAO1-GU1; NBRC 116674, PAO1-GU2; NBRC 116675, PAO1-GU3; NBRC 116676, PAO1-GU4; NBRC 116677, PAO1-GU5; and NBRC 116678, PAO1-GU6.

### Observation of colony morphology and coloration of liquid culture

A glycerol stock of the GU variants was streaked onto an LB plate. The plates were incubated overnight at 37°C, and colony morphology was observed.

A glycerol stock of GU variants was inoculated into LB broth. Bacteria were incubated overnight at 37°C with shaking at 250 rpm, and the liquid culture morphology was observed.

### Growth curve

All isolates were inoculated into LB broth and incubated for 16 h with shaking at 250 rpm. The culture was then diluted with LB broth to an OD_600_ of 0.015. Bacteria were incubated for 36 h with shaking at 50 rpm, and the OD_600_ was measured every 20 min using a TVS062CA (ADVANTEC, Tokyo, Japan). Experiments were conducted in triplicate and independently repeated three or four times.

### Quantification of pyocyanin

The quantification of pyocyanin was conducted based on the absorbance of pyocyanin at 520 nm in an acidic solution, following the method described by Essar et al. (48). The *P. aeruginosa* PAO1 isolates were cultured in LB medium at 37°C overnight, and the overnight cultures were diluted to an optical density at OD_600_ of 0.05 in 12 mL of medium. Subsequently, the cultures were incubated at 37°C and 250 rpm. At 0, 3, 6, 12, and 24 h, an aliquot of the culture was collected and centrifuged at 5,000 × *g* for 1 min. The supernatant was filtered through a 0.22 µm pore-size filter (Merck Millipore, Burlington, MA, USA). The cell-free filtrate was mixed with an equal amount of chloroform, vortexed vigorously for 30 s, and allowed to settle for 10 min. The organic layer was carefully transferred, and 20% of the total volume of 0.2 M HCl was added to obtain a pink to deep-red solution. The absorbance of the aqueous fraction was measured at 520 nm to quantify the pyocyanin levels. The pyocyanin concentration was determined by multiplying the OD_520_ by 17.072. The experiment was conducted in triplicate and independently repeated three times.

### Swimming assay

The swimming assay was performed in 0.25% agar plates (per 1 liter, 2.5 g Bacto agar [Becton Dickinson, Franklin Lakes, NJ, USA], 0.5 g NaCl, and 10 g Bacto peptone [Becton Dickinson, Franklin Lakes, NJ, USA]). A single colony on an LB plate was picked using a sterile toothpick and inoculated onto the surface of a 0.25% agar plate. After overnight incubation at 30°C, the swimming diameter was measured. The experiment was conducted in triplicate and independently repeated three times.

### Twitching assay

Twitching assays were done on LB 1.0% agar plates. A single colony on an LB plate was picked using a sterile toothpick and pierced into the bottom of an LB 1.0% agar plate. After overnight incubation at 37°C, the agar was carefully removed, and the plates were stained with 1% crystal violet for 10 min. The crystal violet was washed with deionized water, and the twitch diameter was measured. The experiment was conducted in triplicate and independently repeated three times.

### Antibiotic susceptibility testing

Antimicrobial susceptibility tests were conducted using premade dry plates (DP-45), according to the manufacturer’s instructions (Eiken Chemical, Tokyo, Japan). The plates contained the following antimicrobial agents: piperacillin, tazobactam, cefepime, ceftazidime, doripenem, imipenem, meropenem, gentamicin, amikacin, tobramycin, levofloxacin, ciprofloxacin, colistin, and aztreonam. Before application, bacterial cultures were adjusted to a density equivalent to 1.0 McFarland standard with saline. Then, 25 µL of the suspension was added to 12 mL of Mueller-Hinton broth (Becton Dickinson, Franklin Lakes, NJ, USA), and 100 µL of this mixture was added to each plate as inoculum. The plates were then incubated for 18 h at 37°C, and the results were visually observed.

### Genome sequencing and bioinformatics analysis

gDNA was extracted using the DNeasy Blood & Tissue Kit (QIAGEN, Venlo, The Netherlands). Whole-genome sequencing was performed using a MiSeq system (Illumina, San Diego, CA, USA). Next-generation sequencing libraries were prepared using the Illumina DNA Prep (Illumina, San Diego, CA, USA). Sequencing was performed using MiSeq Reagent Kits v2 (Illumina, San Diego, CA, USA). Raw reads were quality-filtered using the BBDuK2 plug-in in Geneious Prime 2024.0.7 (Dotmatics, Boston, MA, USA). Each PAO1 genome mutation was identified from reads mapped to *P. aeruginosa* ATCC 15692 (NZ_CP017149.1) using Bowtie2 v2.4.5 (49). Bowtie2 (v2.4.5) was used to build an index from Input.fasta using bowtie2-build. Paired-end reads were then aligned to the indexed reference using the following command: bowtie2 -I 0 -X 900 --sensitive-local -x bowtie2_index -1 forwardReads.fastq -2 reverseReads.fastq -S output.sam. Variant SNPs were identified using the Geneious Prime 2024.0.7 plug-in with a frequency cutoff of 80%.

### Bacteriophage and preparation of phage lysate

*P. aeruginosa* phage PP7 was obtained from the NBRC (catalog number NBRC 105898). For phage lysate preparation, PP7 was plated on a lawn of PAO1-A and incubated overnight at 37°C. Phage buffer (1 mM CaCl_2_, 10 mM MgSO_4_, 68 mM NaCl, and 10 mM Tris-HCl [pH 7.5]) was added to the plate and incubated at room temperature for 5 h. Phage buffer was collected and filtered with a 0.22 µm pore-size filter (Merck Millipore, Burlington, MA, USA).

### Spot assay of PP7 and observation of autoplaquing by GU variants

An overnight culture of the host bacteria was added to LB 0.4% soft agar and poured onto an LB plate. PP7 was serially diluted, and 2 µL of phage was spotted on the lawn of host bacteria. Plates were incubated at 37°C for 7.5 h. To check autoplaquing, the overnight culture of a variant was added to LB 0.4% soft agar and poured onto an LB plate. The plates were incubated at 37°C for 24 h.

### Construction of gene-deletion mutants related to pilus, flagellum, or LPS synthesis

Deletion mutants of PAO1-A were constructed according to the method described by Lesic and Rahme (50), with slight modifications. To construct PAO1∆*galU*::*kan*, upstream and downstream of *galU* were amplified by PCR using galU-up-F/galU-up-R and galU-dw-F/galU-dw-R, respectively. The kanamycin resistance cassette was amplified by kan-F/kan-R using pMW218 (Nippon Gene, Tokyo, Japan) as a template. Nested PCR was carried out using three PCR products to obtain the kanamycin-resistant cassette with upstream and downstream regions of *galU*. To construct other PAO1-A deletion mutants, the kanamycin resistance cassette and upstream and downstream regions of the target gene were amplified by PCR using pMW218 as the template. Primer sets shown in Table S2 were used for PCR. The PCR products were purified using a QIAquick PCR Purification Kit (QIAGEN, Venlo, The Netherlands) and electroporated into electrocompetent bacteria harboring pUCP18-RedS (Invitrogen, Carlsbad, CA, USA). Transformants were selected on LB supplemented with 300 µg/mL of kanamycin. The pUCP18-RedS plasmid was removed by plating on LB plates supplemented with 10% sucrose.

Deletion mutants of *pilT* and *lasR* in the GU variants were constructed using the same method. Primer pairs pilT-del-F/pilT-del-R and lasR-del-F/lasR-del-R were used to construct the GU3Δ*pilT*::*kan* mutant and *lasR* deletion mutants, respectively.

### Transmission electron microscopy

The phage lysates were purified by CsCl density-gradient centrifugation as described by Yamamoto et al. (51), with slight modifications. Four CsCl solutions with densities of 1.3, 1.4, 1.5, and 1.7 g/mL were layered in PET tubes compatible with a himac P40ST swing rotor (Eppendorf, Hamburg, Germany). The phage lysate was applied on top of the CsCl gradient, and the tubes were centrifuged at 22,000 rpm for 3 h at 4°C using a himac CP70E (Eppendorf, Hamburg, Germany). CsCl was removed using Spectra-Por Float-A-Lyzer G2 dialysis devices (Repligen, Waltham, MA, USA) according to the manufacturer’s protocol.

For the observation of cell morphology of bacteria, cells were incubated at 37°C to an OD_600_ of 1.0 in LB medium without NaCl. Two microliters of sample was placed on a carbon-coated copper grid (EM Japan, Tokyo, Japan) and incubated for 2 min at room temperature. Unbound samples were washed with Milli-Q water and negatively stained with uranyl acetate. Phages and bacteria were imaged using a JEM-2100F (JEOL, Tokyo, Japan) and recorded on a Gatan 894 CCD camera (Gatan, Pleasanton, CA, USA).

### Adsorption assay of PP7

PP7 adsorption assays were performed according to Weppelman and Brinton (9) with slight modifications. Bacteria were grown in LB supplemented with 2 mM CaCl_2_ until the bacterial concentration reached approximately 5 × 10^8^ CFU/mL. A 100 mL aliquot of the bacterial culture was centrifuged, and the pellet was resuspended in 5 mL of LB supplemented with 2 mM CaCl_2_. PP7 was added at a multiplicity of infection of 0.001 and incubated at 37°C for 10 min without shaking. At 10 min post-infection, 100 µL of the culture was filtered through a 0.45 µm pore-size filter (Merck Millipore, Burlington, MA, USA). The filters were washed twice, the filtrates were adjusted to a final volume of 10 mL, and the PP7 titer was determined. The PP7 titer in the sample without bacteria was set to 100%, and the percentage of unadsorbed phages was calculated. The experiment was conducted in triplicate and independently repeated three times.

### *pilJ* deletion mutants

To construct *pilJ* mutants, previously reported methods were used with slight modifications (52–54). The primer pair pilH-F and pilJ-del-R was used to amplify the region upstream of *pilJ,* and the primer pair pilJ-del-F and pilK-R was used to amplify the region downstream of *pilJ*. The two PCR products were mixed, and the PCR reaction was performed with pilH-F and pilK-R to obtain the products upstream and downstream of *pilJ*. The product was digested with *EcoR*I and *Hind*III and integrated into pk18msB (Addgene plasmid #177839) (55) or pEX18Ap (synthesized by Invitrogen [Invitrogen, Carlsbad, CA, USA]). The plasmid was electroporated into bacteria. Recombinants were screened by plating on LB plates supplemented with 500 ng/µL kanamycin or 200 ng/µL carbenicillin. Recombinants were incubated overnight in LB without antibiotics to cure the plasmids, and *pilJ* deletion mutants were screened in LB supplemented with 10% sucrose. The curing of the plasmid was confirmed by PCR using the primer pair pK18ms check-F/pK18ms check-R or pEX18T check-F/pEX18T check-R.

### Construction of plasmids

To construct p*pilT*, the ORF of the *pilT* gene was amplified by PCR using the PAO1-A genome as the template and the primers pilT-F and pilT-R. The PCR product was digested with *Mfe*I and *Hind*III and ligated into pUCP18 (Invitrogen, Carlsbad, CA, USA), which was restricted by *EcoR*I and *Hind*III. To construct p*pilJ* and p*lasR*, the ORFs of *pilJ* and *lasR* were amplified by PCR using the PAO1-A genome as the template. For p*pilJ*, pilJ-F and pilJ-R were used as the primer pairs for PCR, and for p*lasR*, lasR-F and lasR-R were used as the primer sets. The PCR products were digested with *EcoR*I and *Hind*III and ligated into pUCP18 restricted by *EcoR*I and *Hind*III.

### Detection of Pf6 and Pf4 by PCR

Genomic DNA of the GU variants was purified using DNeasy Blood & Tissue Kits (QIAGEN, Venlo, The Netherlands), following the manufacturer’s protocol. Twenty nanograms of genomic DNA was used as a template and amplified by PCR. For the detection of Pf6 and Pf4 excision from the genome, Pf6-F/Pf6-R and Pf4-F/Pf4-R were used as primer pairs, respectively. For the detection of RF of Pf6 and Pf4, Pf6att-F/Pf6att-R and Pf4att-F/Pf4att-R were used as primer pairs, respectively. To detect internal regions of the Pf6 and Pf4 prophages, Pf6-in-F/Pf6-in-R and Pf4-in-F/Pf4-in-R were used as primer pairs, respectively. The cycling conditions of PCR were as follows: (i) initial denaturation at 98°C for 3 min; (ii) 25 cycles of 98°C for 10 s, annealing at 65°C for 15 s, and extension at 72°C for 2 min; and (iii) a final extension at 72°C for 2 min. The time required for extension was sufficient to amplify the genome without the Pf6 and Pf4 prophages. However, if Pf6 or Pf4 is integrated into the genomic DNA, the size of the expected PCR product is more than 10 kbp, and the PCR product cannot be amplified under these PCR conditions.

### Isolation of GU7

A glycerol stock of GU5 was streaked onto an LB plate. Twenty colonies were picked and checked for the presence of Pf6 prophages in the genome by colony direct PCR using primers Pf6-F and Pf6-R, as described above. Three colonies were found to lack the Pf6 prophage. These colonies were streaked onto LB plates for single-colony isolation. After three rounds of single-colony isolation, the absence of Pf6 was confirmed by PCR and Sanger sequencing, using the primer sets described above. The genome sequence of GU7 was determined by NGS as described in the previous section, and no additional mutations were identified compared to GU5.

### Data analysis

Means or means ± standard deviations are shown as graphs. The results of the twitching, swimming, and adsorption assays were statistically analyzed using the Kruskal-Wallis test. Statistical significance was set at *P* < 0.05. GraphPad Prism version 10.4.2 (GraphPad Software, San Diego, CA, USA) was used for all data analyses.

## Data Availability

The raw reads from the NGS analysis were deposited in the DDBJ under accession numbers DRR668302 (PAO1-A), DRR668304 (GU2), DRR668305 (GU3), DRR668306 (GU4), DRR668307 (GU5), and DRR668308 (GU6).

## SUPPLEMENTAL MATERIAL

Supplemental Material spectrum.03299-25-s0001.pdf Fig. S1 to S3; Tables S1 and S2.

ASM does not own the copyrights to Supplemental Material that may be linked to, or accessed through, an article. The authors have granted ASM a non-exclusive, world-wide license to publish the Supplemental Material files. Please contact the corresponding author directly for reuse.
