# Supplementary material for: Intra-strain genomic microevolution and phage resistance in Pseudomonas aeruginosa PAO1 laboratory isolates
Source: Microbiol Spectr. 2026 Jul 21;14(9):e03299-25. doi: 10.1128/spectrum.03299-25 (PMC13532354; doi:10.1128/spectrum.03299-25)
Supplement: Supplementary file 1 [file spectrum.03299-25-s0001.pdf]

Fig. S1

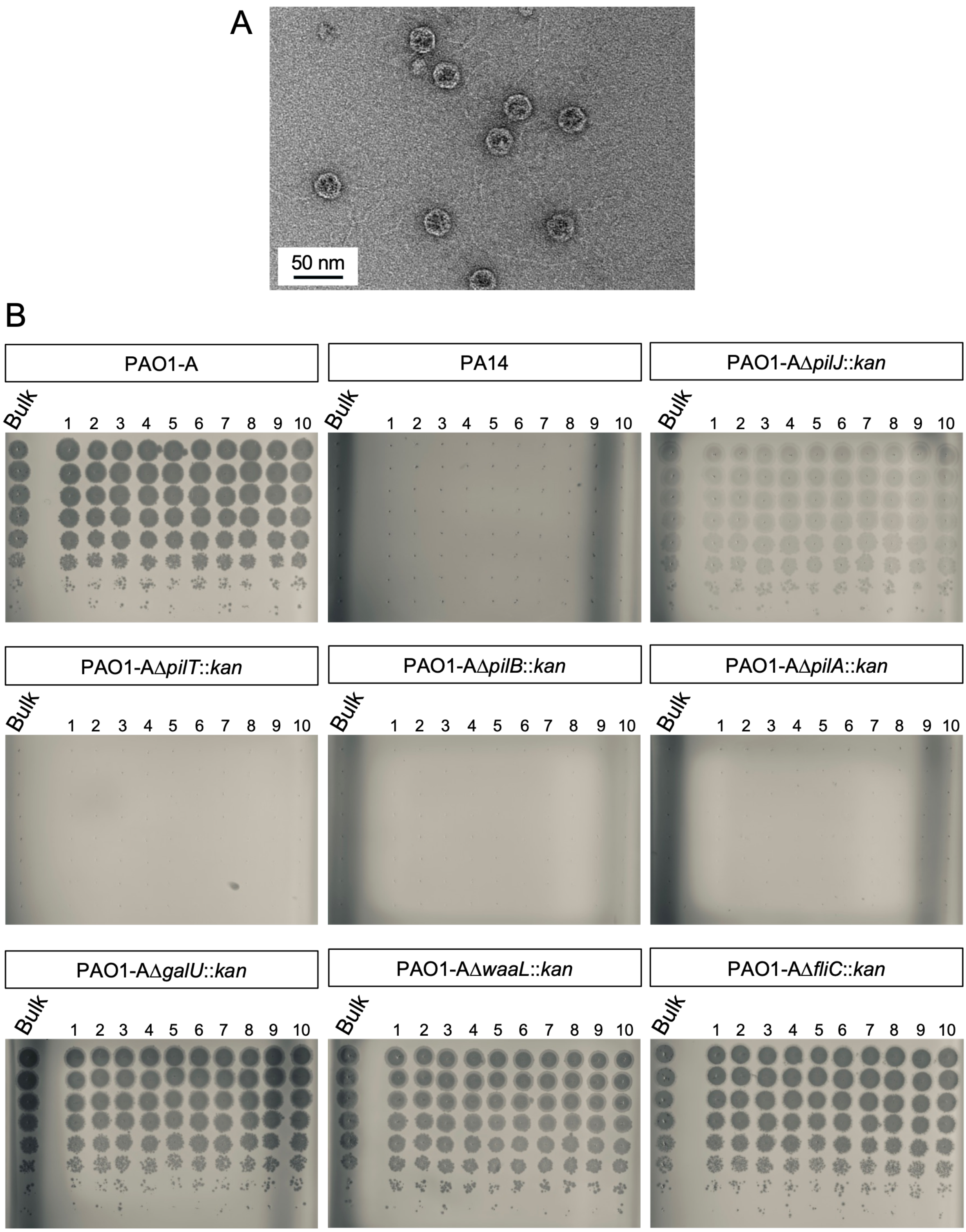

Figure S1. PP7 morphology observed by TEM and pilus requirement for infection.

(A) PP7 lysate was negatively stained with uranyl acetate and visualized by JEM-2100 and recorded on a Gatan 894 CCD camera. Scale bar, 50 nm. (B) An aliquot of the PP7 lysate was plated on a lawn of PAO1-A to obtain single plaques. Ten single plaques were picked and designated 1 to 10. Phages were serially diluted and spotted on lawns of the host bacteria shown in the figure. Plates were incubated at 37°C for 7.5 h. The original PP7 lysate used to obtain single plaques was also spotted as a positive control and is shown as Bulk in the figure.

Fig. S2

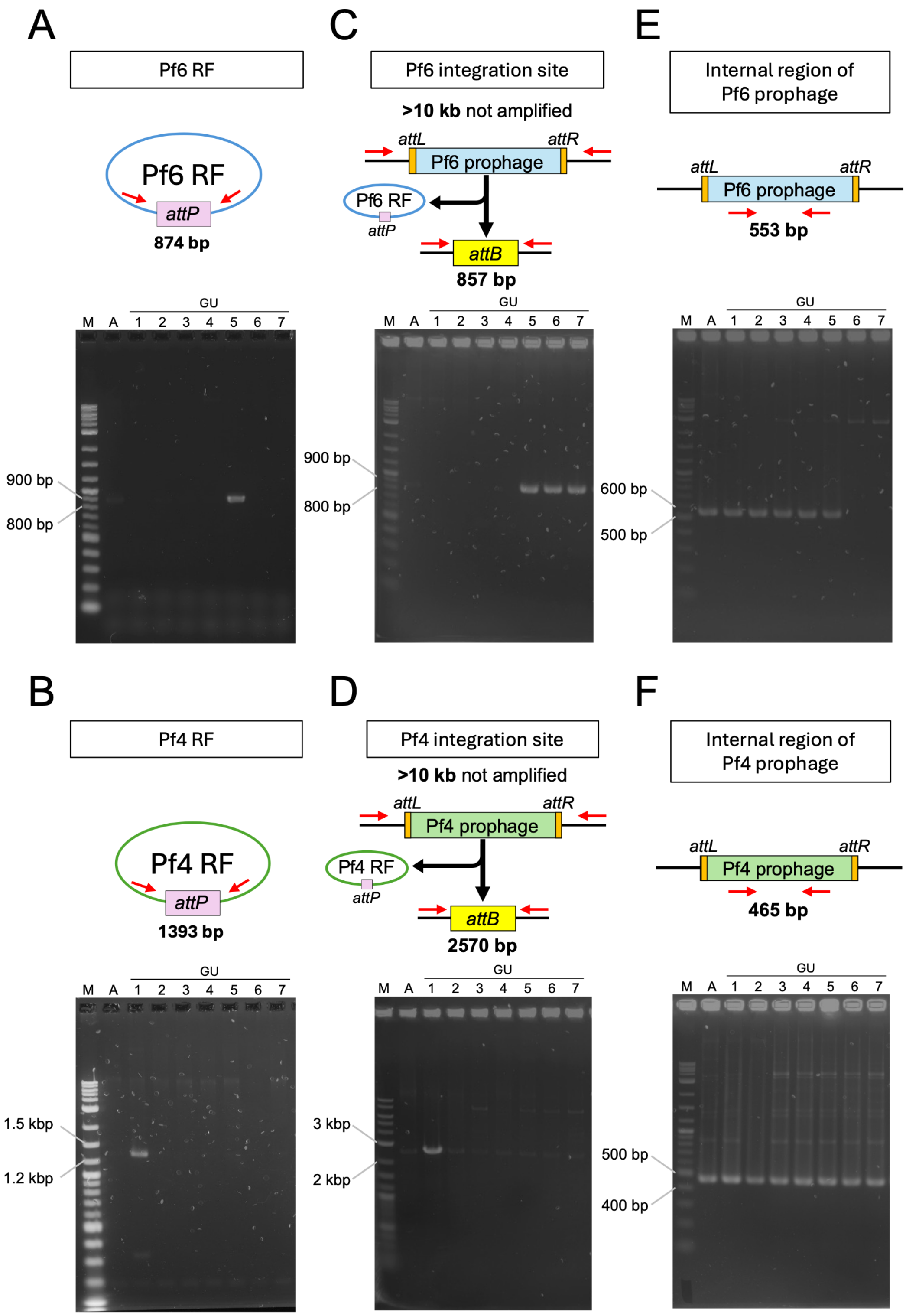

Figure S2. Detection of Pf6 and Pf4 prophages and their replicative forms in the GU variants. (A, B) The RFs of Pf6 and Pf4 were detected by amplifying the *attP* site. (C, D) The integration sites of Pf6 and Pf4, respectively, were examined by PCR using primer pairs, Pf6-2F/Pf6-R for Pf6 and Pf4-F/Pf4-R for Pf4. If Pf6 or Pf4 was integrated at the original site, the expected PCR product would be larger than 10 kb and would not be amplified under the PCR conditions used here. (E, F) To examine the presence of Pf6 and Pf4 prophages in the genome, PCR was performed using primer pairs targeting internal regions of the Pf6 and Pf4 prophages, respectively, as described in Materials and Methods. M, Marker; A, PAO1-A.

Fig. S3

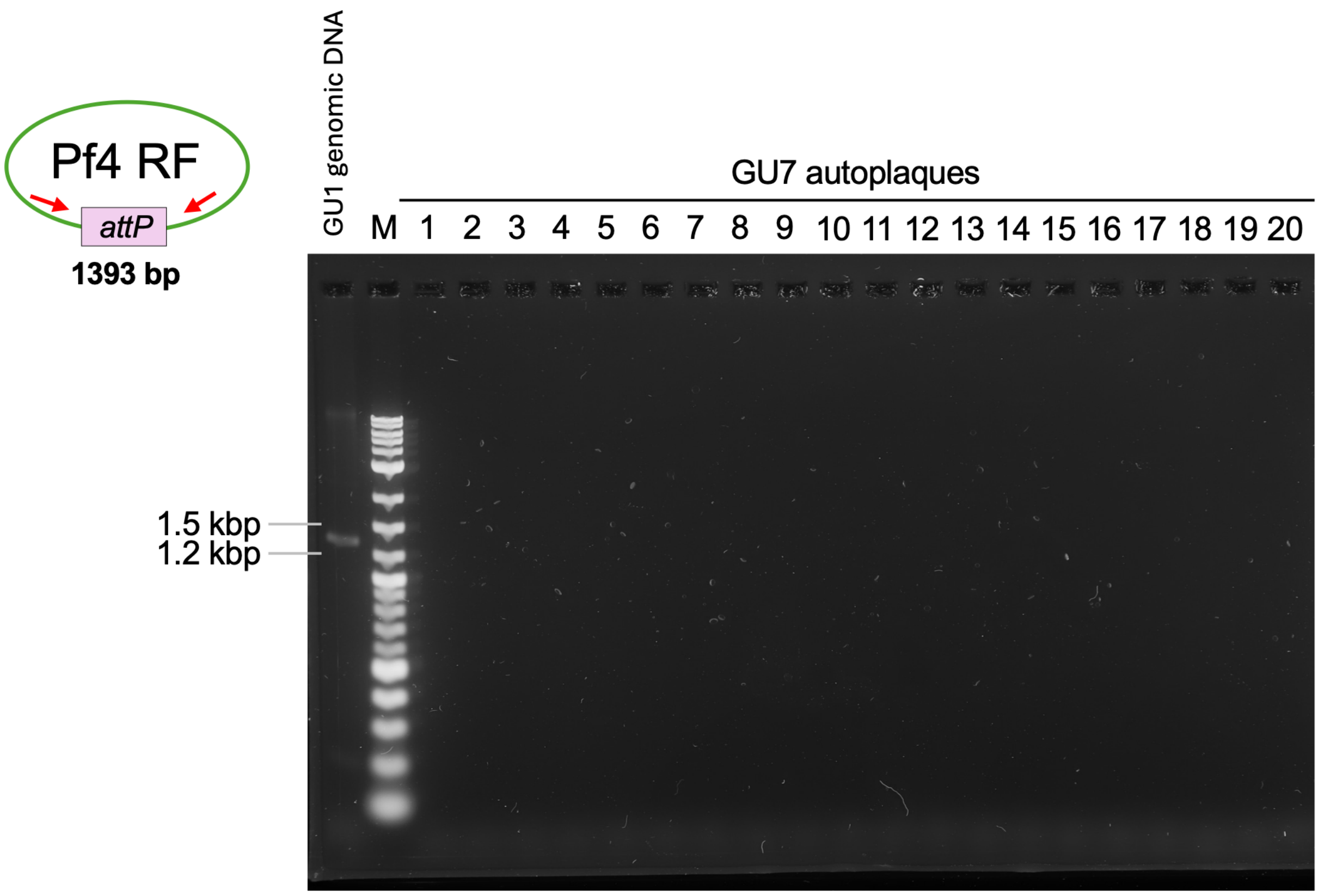

Figure S3. Investigation of autoplaques observed in GU7.

An overnight culture of GU7 was mixed with LB soft agar, poured onto an LB agar plate, and incubated at 37°C overnight. Single autoplaques were picked and suspended in phage buffer. The suspensions were used as templates for PCR targeting the Pf4 RF. PCR was performed as described in Materials and Methods. As shown in Fig. S2B, GU1 was the only variant in which Pf4 RF was detected by PCR; therefore, genomic DNA purified from GU1 was used as a positive control for the PCR reaction.

Table S1

Coverage of whole genome and Pf6 region

	Whole genome	Pf6 region
PAO1-A	108.4x	120.5x
GU2	114.1x	123.1x
GU3	126.7x	146.1x
GU4	136.2x	151.7x
GU5	96.3x	834.9x
GU6	119.6x	2.8x

Table S2

Primers used in this study

Primer	5'-3' sequence	Description
pilT-del-F	GCACCCTGGCTCATCCGGTGTTTTCCTTGTCCGAGACGGCGGCTTTGGCGGCATTCTACCTGCTTGCTTGTAATTGGGGAGTCCCCTTGAGAGTTTTCG	For construction of Δ*pilT*::*kan*
pilT-del-R	GCTCTTGAATCCTAGACGCAGTTCCGGGATTCGGGCGAAGCGCGGCGGATCGGCGCCAGGACGCTCAGAAGAACTC	
pilA-del-F	GATCGTGGCGCGGTTTGATATTTGGCATGGTAAGTGCTTGTTGAGGTTCAGGCGTTAGGCCTATACATAATCAACGGAGAGATTCCCTTGAGAGTTTTCG	For construction of Δ*pilA*::*kan*
pilA-del-R	AGCTAATCCGGCGGCGCGGGGGAGCCTACCATTAGAGGAACCCAATCACAACGGAAGATGGCTAAACAAGCCACCTTCGATCACCCGCTCAGAAGAACTC	
pilB-del-F	TTTGACAGCTGGATTTCCTCGATTTGGCAGCCCCTGTTATCTGCGTTATAAGGCACCTCAATAGCCCTTCTGCGATTCCTTCCCCCCTTGAGAGTTTTCG	For construction of Δ*pilB*::*kan*
pilB-del-R	TCAGTTCGCCCTTGACCTTGGCGCCTTTCTTGTCGGTGCCCTCCCAGATGAAAACGCTGGTTTTTAACGCTTTGTCCGCCATGGACGCTCAGAAGAACTC	
pilJ-del-F-2	AACAGCTTCACCGGTTGGGCCGGTCTGCCGGCCCGTGTCGTGACATGGAAATCGTAATCGTGGTCGGTCCGGGCGGGGGCCAAATCCTTGAGAGTTTTCG	For construction of Δ*pilJ*::*kan*
pilJ-del-R-2	GTGACCAGACGCCGTTCGCCTGCATGCCGTGCCCCTCGTCCAAGGTGTCGACGCGGCGCCCGCCAGGCGGCCGCCGCGCCTATGCCGCTCAGAAGAACTC	
galU-up-F	CGCCAGCCGGACTTCCAG	For construction of Δ*galU*::*kan*
galU-up-R	CTTCGGGGCGAAAACTCTCAAGGCTTGATCATGAGAATCCTTCGAC	
galU-dw-F	TTCTTGACGAGTTCTTCTGAGCGTGAGCCTCGCCGTTCCCG	
galU-dw-R	GCGCTGCCGATTCCCTGAC	
kan-F	CCTTGAGAGTTTTCGCCCCGAAG	
kan-R	CGCTCAGAAGAACTCGTCAAGAA	
waaL-del-F	GTGCTCAAGGCCTATCTCCGCAGCAGCCAGGTCCACTGGACACTCGAGCAGCTCCTCAAGCAACTGCAACTGGATTGAGCAAGGACCTTGAGAGTTTTCG	For construction of Δ*waaL*::*kan*
waaL-del-R	CGGCTTGCCGCTGAAGTACTGGAACGAGAAGACCTTCCGCGGCATTGCGCGGCTGTTTTTCTCCTGATCGCTGATATCGCCGCACCGCTCAGAAGAACTC	
fliC-del-F	ACGCGAATTCTTGGGGCACCTGAGCAAGCAGGCCGAGAGATCGCAAGCTCAGGTAACCGAAATAGGTCCTTTGGAGGAAATCACCCCAGTTGACATAAGC	For construction of Δ*fliC*::*kan*
fliC-del-R	CGTCCATTTCTCATGCCTCGCAAAGGGAAAAGGGAGGAAGGGTCACCCCTTCCTCCCAGTACCGCGTGAGTGACCGTTCCCGGGCTTGTTAGGTGGCGGT	
lasR-del-F	TCGGACTGCCGTACAACGTGCCGGATATCGGGTGCCGAATCCATATTTGGCTGATTGGTTAATAGTTTAAGAAGAACGTAGCGCTCCTTGAGAGTTTTCG	For construction of Δ*lasR*::*kan*
lasR-del-R	CTGCCCTTCCCTATATATCTGCTTTTGCATTTCTATATAGAAGGGCAAATTACCGATCGCCAGCTCGCCGACCTGAGAGGCAAGACGCTCAGAAGAACTC	
pilH-F	AAAGAATTCGGTCACCAGGTACTCAAGGC	For construction of *pilJ* deletion mutant
pilJ-del-R	GTCTGCGAGGTGATCTCCTGGTTCGACTGGGTGTTGAGGT	
pilJ-del-F	ACCTCAACACCCAGTCGAACCAGGAGATCACCTCGCAGAC	
pilK-R	AAAAAGCTTCCTTACGGGTAAAGGCCAGG	
pK18ms check-F	GCCTGGTATCTTTATAGTCCTGTCGG	For confirming the cure of pK18ms
pK18ms check-R	CAGAGCAGCCGATTGTCTGTTG	
pEX18T check-F	GTGCTGCAAGGCGATTAAGTTGG	For confirming the cure of pEX18T
pEX18T check-R	GCTCACTCATTAGGCACCCCAG	
pilT-F	AAACAATTGTACCTGCGCCCTATGGAAGTG	For constution of p*pilT*
pilT-R	GGGAAGCTTTCAGAAGTTTTCCGGGATCTTCG	
lasR-F	CCCGGAATTCCGAACTGGAAAAGTGGCTATGTCG	For constution of p*lasR*
lasR-R	GGGAAGCTTTCAGAGAGTAATAAGACCCAAATTAACGGC	
pilJ-F	CCCGGAATTCACGTCGCCGTATAAACAGCTTCACCG	For constution of p*pilJ*
pilJ-R	GGGAAGCTTTCAGGCCTGCTCCACGCCCTC	
Pf6-F	CTCAGTTCTGGATGAATCTCCAGAGTG	To detect Pf6 excision
Pf6-2F	ATCAATGACCAGTGGCGTGTC	
Pf6-R	GCAAGGACTACATCGTCAAGGACG	
Pf6att-F	GGTAGACACGCTGTAGTCACTTTGTAG	To detect RF of Pf6
Pf6att-R	TTCCGATGCAGCGAAGGGGG	
Pf6-in-F	TGCTCTCCATGCACCACCACC	To detect internal region of Pf6 prophage
Pf6-in-R	TTCCGATGCAGCGAAGGGGG	
Pf4-F	AGTGGCGGTTATCGGATGAC	To detect Pf4 excision
Pf4-R	TCATTGGGAGGCGCTTTCATC	
Pf4att-F	CCCCCAAAAAGCAAAGCCC	To detect RF of Pf4
Pf4att-R	AACATTAAGCCCCAGCAGCT	
Pf4-in-F	AGGCTGCGGGGGTCGACAG	To detect internal region of Pf4 prophage
Pf4-in-R	TTATTCTGGCTGAGCGAACCTCC	
